# Comparison of non-operative versus operative management of resectable colorectal cancer in elderly patients: study protocol for a systematic review

**DOI:** 10.1186/s13643-022-01949-w

**Published:** 2022-04-25

**Authors:** Richard Hu, Rajajee Selvam, Husein Moloo, Lara Williams, Isabelle Raiche, Reilly Musselman

**Affiliations:** 1 Department of Surgery, University of Ottawa, Ottawa, ON Canada; 2 The Ottawa Hospital, Civic Campus, 305-737 Parkdale Ave, Ottawa, ON K1J 4E9 Canada

## Abstract

**Background:**

In the 2021 Statistics Canada census, 18.5% of the Canadian population were senior (65 years and older), among those 1.7 million (4.5%) were aged 80 years and older. Colorectal cancer (CRC) is the third most common cancer in both men and women, with its highest incidence rate in septu- and octogenarians. As clinicians encounter a growing number of very elderly patients (80 years and older) with resectable colorectal cancer, justifying major surgery in a comorbid population with limited life expectancy is difficult. Therefore, this study aims to systemically review the available literature to compare non-operative management to surgical resection with respect to overall survival and quality of life.

**Method:**

We designed and registered a study protocol for a systematic review. We will include all patients above the age of 80 with resectable colorectal cancer. We will search MEDLINE, EMBASE, and the Cochrane Database of Controlled Trials from January 2000 onwards. We will include randomized, non-randomized controlled trials and observational studies comparing non-operative versus operative management of resectable colorectal cancer in elderly patients. The primary outcomes will be overall survival and mortality. Secondary outcomes will include quality of life, and health services/ resources utilization (e.g., treatments, change of level of care…). Two reviewers will independently screen all citations, full-text articles, and abstract data. Potential conflicts will be resolved through discussion. The study methodological quality (or bias) will be appraised using the ROB-2 and ROBIN-I tools. If feasible, we will conduct random effects meta-analysis. Additional analyses will be conducted to explore the potential sources of heterogeneity (e.g., study design and methodological quality).

**Discussion:**

This systematic review will synthesize the existing data on the management of colorectal cancer in the very elderly patients, and identify the gap in the literature for potential future research. More specifically, we aim to streamline non-operative outcome data on resectable colorectal cancers to aid clinicians’ decision-making with respect to survival outcomes and quality of life. The results of this study will be of interest to multiple audiences including patients, their families, caregivers, healthcare professionals, and policy makers. Results will be published in a peer-reviewed journal.

**Supplementary Information:**

The online version contains supplementary material available at 10.1186/s13643-022-01949-w.

## Introduction

In the 2021 Statistics Canada census, 18.5% of the Canadian population were senior (65 years and older), among those 1.7 million (4.5%) were aged 80 years and older [1]. Globally, the number of persons aged 80 years or over is projected to increase more than threefold between 2017 and 2050, rising from 137 million to 425 million [2]. Meanwhile, it is estimated that 6.25% develop colorectal cancer (CRC) during their lifetime [3], with its incidence rising steeply from around the age of fifty to reach the highest rates in septu- and octogenarians [4]. With an aging population, combined with increased incidence in the elderly, the prevalence of CRC will continue to rise in this population.

Treatment algorithms for CRC are well established in today’s literature. However due to increased co-morbidity and frailty concerns, in addition to higher postoperative morbidity and mortality rates than their younger counterparts, treatment decisions in the elderly population have become more complex [5, 6]. To address this issue, the International Society of Geriatric Oncology (SIOG) 2013 task force performed an overview of recent data on epidemiology and geriatric of assessments of CRC patients [7]. As part of the overall conclusions and recommendations, they urged the development of a separate treatment guideline for elderly patients with CRC as they identified a gap in the literature for this special population. No systematic reviews have been done to investigate the outcomes of those who decide against surgery. One systematic review on this topic was published in the Lancet in 2000 looking at surgical outcomes in the elderly with colorectal cancer, reporting a rate of non-operative of 21% in the group 85 years and older [8].

As clinicians encounter a growing number of very elderly patients (80 years and older) with resectable colorectal cancer, justifying major surgery in a comorbid population with limited life expectancy is difficult. In fact, there is a paucity of literature around the natural disease progression without surgical intervention to guide the treatment discussions. A limited number of small series, observational studies published in recent years compared these two treatment arms in attempt to answer this question [9–11], with early results favoring operative management. However, these data have not been synthesized. In addition, with decreased physiologic reserve in elderly patients [12], little is known in respect to the impact of major colorectal surgery on their postoperative quality of life and functional status. Therefore, we aim to systemically review the available literature to compare non-operative management of resectable colorectal cancer to surgical resection in patients 80 years and older to assess for overall survival and quality of life.

## Methods

This systematic review will be conducted based on a review protocol registered with the International Prospective Register of Systematic Reviews (PROSPERO registration CRD42020199509) and is being reported in accordance with the reporting guideline provided in the Preferred Reporting Items for Systematic Reviews and Meta-Analyses Protocols statement (see checklist in Additional file 1) [13].

### Eligibility criteria

The study selection criteria have been established according to the PICOS (Population – Intervention – Comparator – Outcomes – Study design)

- Population: Elderly population, 80 years and older, with surgically resectable (with intent to cure) colorectal cancer
- Intervention: Surgical resection with curative intent, excluding palliative procedures such as bypass or diverting stomas, including metastatic tumors provided surgery is with curative intent (Table 1)

**Table 1:** Included and excluded procedures in the intervention group

Included procedures (both laparoscopic and open)	Excluded procedures
- Left hemicolectomy - Right hemicolectomy including extended right hemicolectomy - Segmental colectomy - Total abdominal colectomy - Total proctocolectomy - Low anterior resection - Proctectomy - Trans-anal resection of rectal cancers - Metastasectomy with curative intent - Other (any surgical resection with curative intent)	- Diverting ostomy without tumor resection - Palliative non-oncological resection - Bypass surgery - Stenting

- Comparison: Non-operative management/palliation (Table 2)

**Table 2:** Included treatment in the non-operative group

Included treatments	Excluded treatments
- Watchful waiting - Palliation/symptom control - Diverting ostomy without tumor resection - Palliative non-oncological resection - Bypass surgery - Stenting - Palliative chemotherapy - Palliative radiotherapy	- All procedures listed in Table 1 - Other (all treatment modalities with intent to cure)

- Primary outcomes: the primary outcome will be overall survival and mortality. This will be calculated in months from the time of diagnosis to the time of death. We will aim to capture both overall survival and cancer-specific survival in operative and non-operative patients. Alternatively, this outcome can be reported in percent survival at 1-, 3-, and 5-year dependent on the data available.
- Secondary outcomes:
  - ° Quality of life after diagnosis: this will likely be patient-reported outcomes through surveys or questionnaires during the study period. This aims to assess patient’s physical, mental and spiritual health after their treatment decision. We have not imposed a strict definition to “quality of life” used in each respective study. We will record which domain of the quality of life was recorded in each study.
  - ° The need for future treatments after treatment decision (e.g., palliative surgery, emergency surgery, radiation). This pertains mostly to the non-operative group to explore if these patients eventually end up requiring rescue procedures.
  - ° Resource utilization after treatment decision to evaluate the economic impact of both operative and non-operative management.
  - ° Change of level of care (e.g., functional decline after treatment requiring assisted living, long-term care home, rehabilitation). This aims to elucidate the impact of surgery or the decision to not undergo surgery on elderly patients’ functional status.
  - ° Postoperative complications: infection, bleeding, anastomotic complications, cardio-pulmonary complications, transfer to intensive care unit, death.
- Study designs: We will include randomized, non-randomized controlled trials, and observational studies (such as prospective and retrospective cohort studies). Cross-sectional studies, case series, and case reports will be excluded. We will also exclude commentaries, letters, and review articles.

### Search strategy and data sources

We will search MEDLINE, EMBASE, and the Cochrane Database of Controlled Trials from 2000 and onwards using a predetermined search strategy developed with the assistance of a health information specialist with expertise in systematic reviews and clinical expert in the field of colorectal surgery (R.M). The search strategy will be comprised of Medline subject headings and key words. A draft search strategy for MEDLINE is available in Additional file 2. The search strategy will be peer-reviewed by a second health information specialist using the PRESS framework to ensure robust capture [14]. We will also search gray literature to include abstracts presented at relevant society meetings from the past three years and ongoing key websites (ClinicalTrials.gov, etc.) to explore ongoing and upcoming studies relevant to our review.

### Study selection and data collection

Two authors (R.H and R. S) will complete abstract screening independently and in duplicate using Mendeley systematic review manager software. Full-text screening will be completed in duplicate by two authors (R.H and R.S). To ensure consistent application of selection criteria, the two reviewers (R.H and R.S) will carry out a pilot exercise comparing their study selection. This will be done during each stage of selection, after a review of the first 50 abstracts at stage 1 and the review of the 20 full texts at stage 2. All disagreements will be settled by a third-party reviewer (R.M). The study selection process will be summarized in a PRISMA flow chart.

### Data extraction

Two authors (R.H and R.S) will complete data extraction independently and in duplicate using a standardized data extraction form implemented in Excel. A pilot extraction exercise of three studies will be performed to ensure consistency in the approach between reviewers. Data elements to be collected will include those related to basic publication characteristics (including year, journal, authorship list, and country), study methods (including design and elements necessary for risk of bias appraisal), population studied (enrollment criteria and key demographic measures including age, gender, BMI, comorbidities, ASA, preoperative nutritional status, anemia, frailty index, smoking, TNM stage of the cancer, tumor location), intervention compared (non-operative—observation and palliative procedures, chemotherapy, radiation therapy, surgical intervention—laparoscopic or open) and outcome data (survival/life expectancy, quality of life, need for future treatment, change in care needs, postoperative morbidity and mortality). Binary outcomes will be collected as *n* (%) and continuous outcomes will be collected as mean (SD).

### Risk of bias assessment

All included studies will be assessed for risk of bias using the Risk of Bias 2.0 tool for randomized controlled trials and ROBINS-I for non-randomized studies [15, 16]. The assessments will be performed both at the outcome and study level. Two authors (R.H and R.S) will complete risks of bias assessments using the abovementioned criteria. Disagreements will be settled by a third-party reviewer (R.M). Findings will be narratively summarized in the text of the final study with full assessments available in an Additional file 3. Sensitivity analyses based upon findings from the assessments (e.g., focused upon high methodologic quality studies) will be considered in the event that studies are pooled for analysis.

### Data synthesis

Key characteristics of the included studies will be summarized and presented in tables. The literature search for this review may identify both comparative studies and single group studies. Single group studies will be focused upon reporting experience with either operative or non-operative treatment, whereas comparative studies will be assessed and analyzed separately from the latter. Depending on the available literature after screening, the data may be analyzed using qualitative/narrative synthesis, or quantitative synthesis.

If a qualitative/ narrative synthesis approach is chosen after reviewing the available studies, these studies will be synthesized to explore heterogeneity descriptively rather than statistically. Results of these studies will be presented under the forms of structured narratives or summary tables following the guidance provided by the SWiM reporting guidelines for synthesis without meta-analysis [17]. The data from each study will be used to build evidence tables of an overall description of included studies.

If enough data can be pooled to formally perform a quantitative synthesis (in the event of uncertainty, a statistician will be consulted), a random effect model will be used to perform the meta-analysis. We also anticipate our main outcome to be dichotomous or time to event measures. For dichotomous outcomes, odds ratios or risk ratios with associated 95% confidence intervals will be used as summary measures. For time to event measures, hazard ratios will be extracted from each study or calculated from the available data. Forest plots will then be used to present the outcomes of individual studies with corresponding 95% confidence intervals and the pooled estimate of effect across all studies with 95% confidence intervals.

The heterogeneity of effect sizes (i.e., statistical heterogeneity) across included studies will be examined using the Cochrane Q and the *I*^2^ statistic. We will use categories of low (0–25%), moderate (25–50%), and substantial (50–100%) to interpret the *I*^2^ statistic. If there is sufficient data and homogeneity across studies, a meta-analysis will be conducted for the outcomes of interest.

### Additional analyses

If sufficient data exist, planned subgroup analyses include stratifying patients by age (80-85, 85-90, 90+), Charleston comorbidity index, ASA score (1–5), cancer stage (1–4), metastatic versus non-metastatic disease, location of the tumor (left colon, right colon, transverse colon, rectum), emergency versus elective surgery, laparoscopic versus open, frailty index, pre-operative anemia, and smoking.

Sensitivity analyses of studies judged to be at low risk of bias will be performed if sufficient data are available.

### Meta-biases

To minimize the potential for publication bias, abstracts without full-text publications will be considered for inclusion, provided they meet inclusion criteria and report on at least one outcome of interest. If individual data is not available, we will attempt to contact corresponding authors to obtain the necessary data. Random-effects models will be used to mitigate the possibility of small sample bias. Funnel plots and Egger’s regression tests will be used to identify possible publication and small study bias if 10 or more studies are included.

### Confidence in cumulative evidence

If a meta-analysis is performed, The GRADE approach will be used to rate the quality of evidence [18]. This framework involves an assessment of risk of bias, consistency, directness, precision, and risk of reporting bias for each studied outcome. A table presenting a summary of the main findings will be generated using the *GRADEpro* software.

## Discussion

The goal of this review is two folds: synthesize the existing data on the management of colorectal cancer in the very elderly patients, and identify the gap in the literature for potential future research. A systematic review published in *Lancet* in 2000 pooled 34,194 patients from 28 independent studies reported that despite the increased incidence of postoperative morbidity and mortality with advancing age, cancer-specific survival showed little difference between the studied age groups (65–74, 74–84, 85+), suggesting the likely benefit of surgical resection regardless of patients age [8]. However, most data were retrospective, with an inherent risk of selection bias. The very elderly patients chosen for surgical resection were likely the result of a careful selection of physiologically fitter patients by the surgical team. This review also had no data on patients’ postoperative functional status and quality of life, an important factor to be considered when offering an elderly patient surgical treatment, and it did not include any data on non-operative management for very elderly patients with the curative disease.

Therefore, there is a need to gather more recent data comparing operative to non-operative management (2000 and onward), with the goal of determining the role of surgical resection in the very elderly patients with colorectal cancer. In addition to survival outcomes, this review will also focus on the impact of surgery on patient quality of life and functional outcomes. Ultimately, this review aims to streamline information to help clinicians with treatment decisions and facilitate discussions with elderly patients using evidence-based findings.

There is an expected paucity of data in this field, which the authors believe will pose certain challenges and limitations to this review. For example, we expect a lack of outcome data with regards to non-operative management of resectable disease as these patients may not require a rigorous surgical follow-up, especially elderly patients who choose the comfort care approach in the community. We also envision large degrees of heterogeneity with retrospective data and suboptimal study designs. As such, we may encounter difficulty performing quantitative analyses such as a meta-analysis. In addition, outcomes such as quality of life and change of level of care lack widely recognized assessment tools, which will render comparisons problematic. Ultimately, despite these challenges, this review will serve to synthesize the existing data and potentially help identify the gap in literature to lay the groundwork for future research.

Any amendments made to this protocol when conducting the review will be outlined in PROSPERO and reported in the final manuscript. Our goal is to have our results disseminated through conference presentations and publication in a peer-reviewed journal.

## Supplementary Information

**Additional file 1.** PRISMA-P 2015 Checklist.

**Additional file 2.** Search strategy.

**Additional file 3.** Risk of Bias Tool.
