# Supplementary material for: Comparison of non-operative versus operative management of resectable colorectal cancer in elderly patients: study protocol for a systematic review
Source: Syst Rev. 2022 Apr 25;11:77. doi: 10.1186/s13643-022-01949-w (PMC9040201; doi:10.1186/s13643-022-01949-w)
Supplement: Supplementary file 2 — Additional file 2. Search strategy. [file 13643_2022_1949_MOESM2_ESM.docx]

Search strategy

1 exp Colorectal Neoplasms/ (237059)

2 ((colorect* or colo-rect* or colon* or rect*) adj2 (cancer* or tumo?r or neoplasm* or carcinoma*)).tw. (487601)

3 ((colorect* or colo-rect* or colon* or rect*) and (cancer* or tumo?r or neoplasm* or carcinoma*)).kf. (38522)

4 1 or 2 or 3 (570080)

5 *"Aged, 80 and over"/ (1935)

6 ("80" adj2 age*).tw. (50462)

7 ("90" adj2 age*).tw. (16798)

8 ("80 years" or "90 years").tw. (101493)

9 (octogenarian* or nonagenarian* or centenarian*).tw,kf. (15299)

10 ("very elderly" or "oldest old").tw. (55644)

11 elderly patient*.tw. (186559)

12 elderly.ti,kf. (295913)

13 or/5-12 (527073)

14 4 and 13 (8712)

15 Life Expectancy/ or life expectancy.tw,kw. (114208)

16 Mortality/ or Hospital Mortality/ or mortality.tw. (2244212)

17 outcome*.ti. (897064)

18 Prognosis/ or prognos*.tw,kf. (2044720)

19 Survival Rate/ or survival.tw,kf. (2575275)

20 *"Quality of Life"/ or "quality of life".tw,kf. (839783)

21 or/15-20 (6845000)

22 14 and 21 (5081)

23 exp Cohort Studies/ (2737152)

24 (cohort or retrospective* or prospective*).tw,kw. (4646285)

25 Cross-Sectional Studies/ (557962)

26 (cross sectional adj2 stud*).tw. (453570)

27 randomized controlled trial.pt. (1003706)

28 controlled clinical trial.pt. (185258)

29 randomized.ab. (1730761)

30 placebo.ab. (789984)

31 clinical trials as topic.sh. (224879)

32 randomly.ab. (1028794)

33 trial.ti. (846392)

34 Cross-Sectional Studies/ (557962)

35 cross section*.tw. (905307)

36 case series.tw. (180356)

37 or/23-36 (9641546)

38 22 and 37 (2901)

39 38 use medall (1060)

40 exp *colorectal cancer/ (276897)

41 ((colorect* or colo-rect* or colon* or rect*) adj2 (cancer* or tumo?r or neoplasm* or carcinoma* or adenocarcinoma*)).tw. (503444)

42 40 or 41 (558510)

43 *very elderly/ (1555)

44 (octogenarian* or nonagenarian* or centenarian*).tw. (15155)

45 ("elderly" or "oldest old").tw. (643963)

46 very old.tw. (65163)

47 ("80" adj2 age*).tw. (50462)

48 ("90" adj2 age*).tw. (16798)

49 ("80 years" or "90 years").tw. (101493)

50 or/43-49 (811353)

51 42 and 50 (10886)

52 life expectancy/ (68201)

53 life expectancy.tw. (80422)

54 hospital mortality/ or cancer mortality/ or mortality/ (999388)

55 mortality.tw. (1961349)

56 outcome*.ti. (897064)

57 prognosis/ or cancer prognosis/ (1282153)

58 prognosis.tw. (999107)

59 survival rate/ (432614)

60 survival.tw. (2443764)

61 *"quality of life"/ (193382)

62 (quality of life or qol or hrqol).tw. (822234)

63 or/52-62 (6707828)

64 51 and 63 (6119)

65 random*.tw. or placebo:.mp. or double-blind:.mp. (4205637)

66 randomized controlled trial/ (1118009)

67 cohort analysis/ (850463)

68 longitudinal study/ (276071)

69 prospective study/ (1150423)

70 (cohort or retrospective* or prospective*).tw. (4626823)

71 cross-sectional study/ (686656)

72 cross section*.tw. (905307)

73 case study/ and series.tw. (77279)

74 case series.tw. (180356)

75 or/65-74 (9698936)

76 64 and 75 (3229)

77 76 use emczd (1766)

78 exp Colorectal Neoplasms/ (237059)

79 ((colorect* or colo-rect* or colon* or rect*) adj2 (cancer* or tumo?r or neoplasm* or carcinoma*)).tw,kw. (497382)

80 78 or 79 (575286)

81 *"Aged, 80 and over"/ (1935)

82 ("80" adj2 age*).tw. (50462)

83 ("90" adj2 age*).tw. (16798)

84 ("80 years" or "90 years").tw. (101493)

85 (octogenarian* or nonagenarian* or centenarian*).tw,kw. (15605)

86 ("very elderly" or "oldest old").tw. (55644)

87 elderly patient*.tw. (186559)

88 elderly.ti,kw. (318965)

89 or/81-88 (545143)

90 80 and 89 (8990)

91 Life Expectancy/ or life expectancy.tw,kw. (114208)

92 Mortality/ or Hospital Mortality/ or mortality.tw. (2244212)

93 outcome*.ti. (897064)

94 Prognosis/ or prognos*.tw,kw. (2061642)

95 Survival Rate/ or survival.tw,kw. (2586675)

96 *"Quality of Life"/ or "quality of life".tw,kw. (853900)

97 or/91-96 (6870345)

98 90 and 97 (5267)

99 98 use cctr (568)

100 39 or 77 or 99 (3394)

101 remove duplicates from 100 (2543)

**102 101 use medall (1055) Medline**

**103 101 use emczd (1073) Embase**

**104 101 use cctr (415) Cochrane**
