# Supplementary figures and images for: Comparison of non-operative versus operative management of resectable colorectal cancer in elderly patients: study protocol for a systematic review
Source: Syst Rev. 2022 Apr 25;11:77. doi: 10.1186/s13643-022-01949-w (PMC9040201; doi:10.1186/s13643-022-01949-w)

Risk of bias tools

ROBINS-I


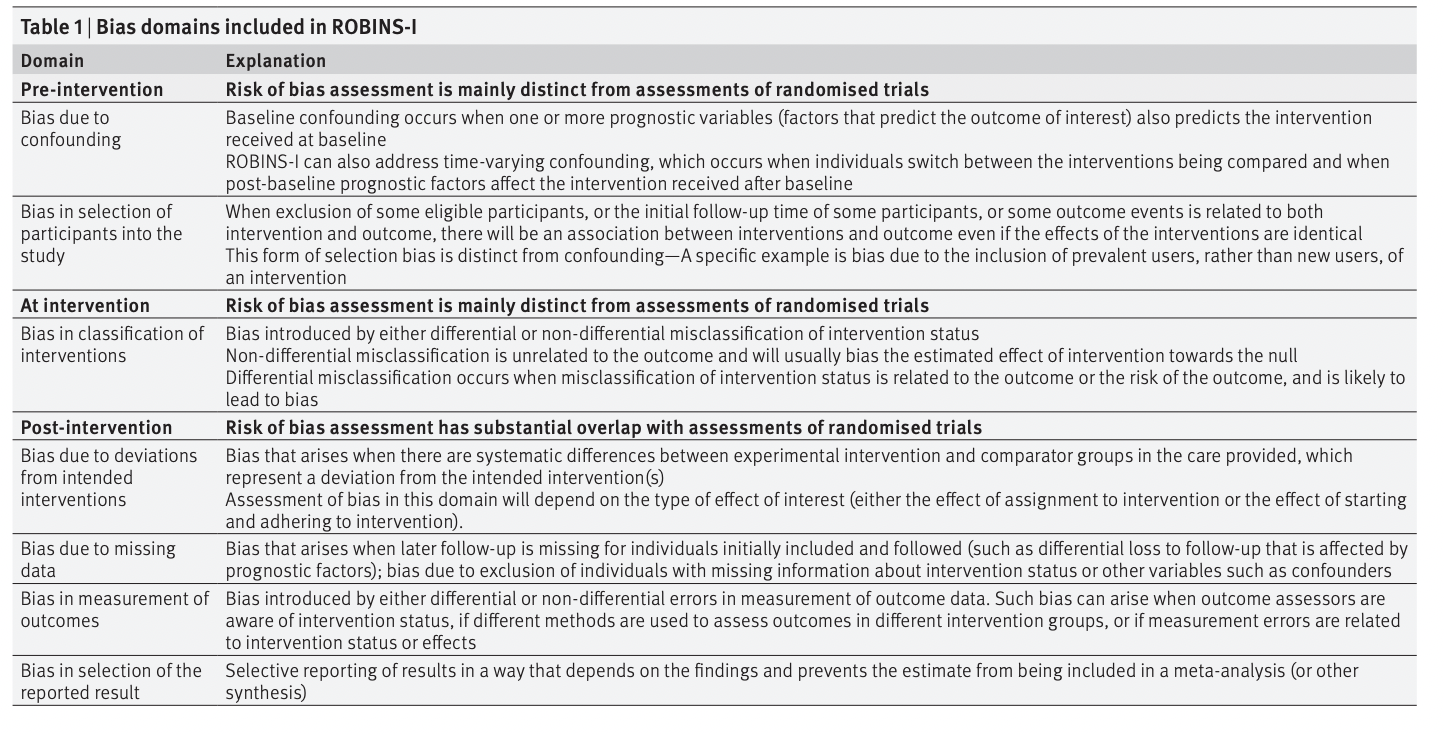


Risk of Bias 2.0 tool


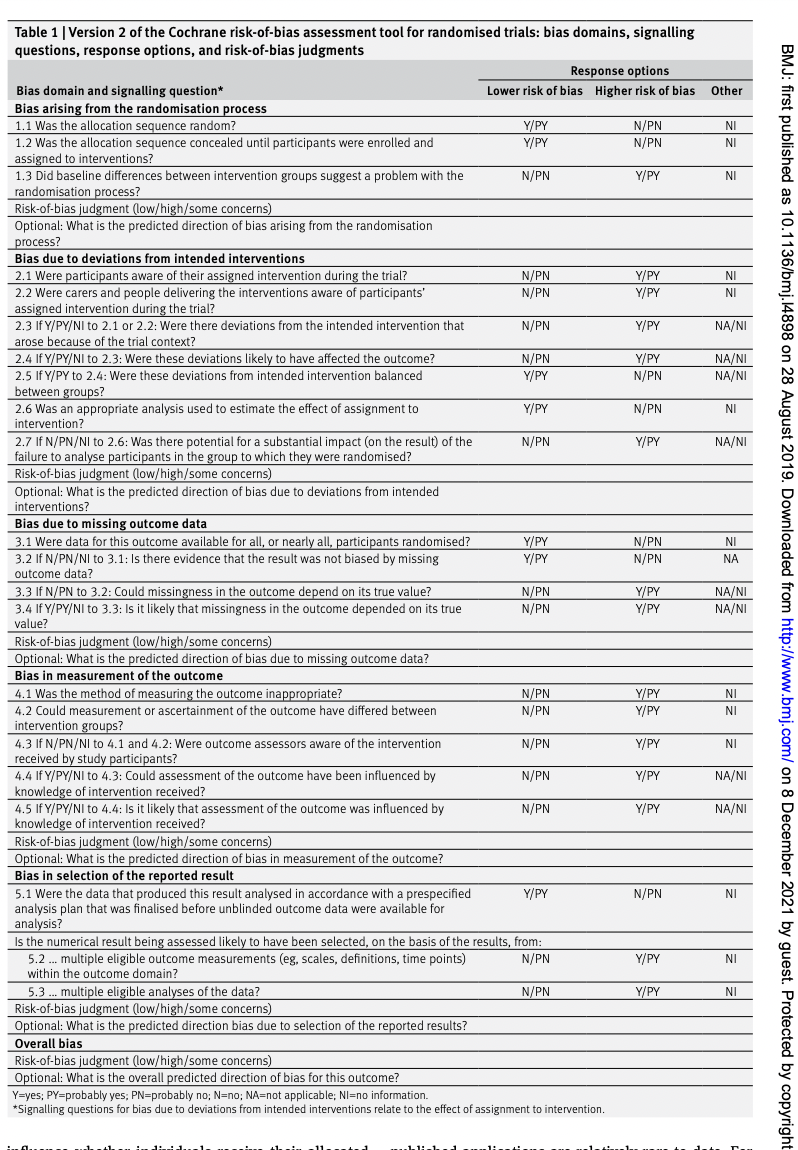

Supplement: Supplementary file 3 — Additional file 3. Risk of Bias Tool. [file 13643_2022_1949_MOESM3_ESM.docx]
